# Meropenem-Vaborbactam in the Treatment of Acute Bacterial Infections

**DOI:** 10.3390/jcm8101650

**Published:** 2019-10-11

**Authors:** Chih-Cheng Lai, Chi-Chung Chen, Hung-Jen Tang

**Affiliations:** 1 Department of Internal Medicine, Kaohsiung Veterans General Hospital, Tainan Branch, Tainan 71051, Taiwan; 2 Department of Medical Research, Chi Mei Medical Center, Tainan 71004, Taiwan; 3 Department of Food Science, National Chiayi University, Chiayi 60004, Taiwan; 4 Department of Health and Nutrition, Chia-Nan University of Pharmacy and Science, Tainan 71710, Taiwan; 5 Department of Medicine, Chi Mei Medical Center, Tainan 71004, Taiwan

**Keywords:** meropenem-vaborbactam, acute bacterial infection, complicated urinary tract infection/acute pyelonephritis

## Abstract

This study reports the integrated analysis of two phase III studies of meropenem-vaborbactam in the treatment of acute bacterial infections. Targeting Antibiotic Non-Susceptible Gram-Negative Organisms (TANGO) I compared the clinical efficacy and tolerability of meropenem-vaborbactam and piperacillin-tazobactam in the treatment of complicated urinary tract infection (cUTI)/acute pyelonephritis (APN). TANGO II compared the effect and safety of meropenem-vaborbactam and best-available therapy in the treatment confirmed/suspect carbapenem-resistant Enterobacteriaceae infections. The clinical cure rates at end of treatment (EOT) and test of cure (TOC) among the meropenem-vaborbactam group were non-inferior to those of the control group (at EOT, 92.5% versus 89.3%, risk ratio (RR) 1.27, 95% CI 0.64–2.50; at TOC, 86.2% versus 81.7%, RR 1.37, 95% CI 0.62–3.01). Meropenem-vaborbactam was non-inferior to comparators for microbiological eradication at EOT and TOC (at EOT, 93.3% versus 88.3%, RR 1.21, 95% CI 0.74–1.97; at TOC, 66.5% versus 59.9%, RR 1.12, 95% CI 0.97–1.30). In the subgroup of patients with cUTI/APN, meropenem-vaborbactam had similar overall success rate to the control group at EOT (RR 1.05, 95% CI 1.01–1.09) and at TOC (RR 1.05, 95% CI 0.93–1.19). Meropenem-vaborbactam had a similar risk of treatment-emergent adverse events, events leading to discontinuation of the study drug, any serious adverse events, life-threatening adverse events, drug-related adverse events, and risk of death to comparators. In conclusion, meropenem-vaborbactam was noninferior to comparators for clinical cure and microbiological eradication in the treatment of acute bacterial infection, particularly cUTI/APN, and meropenem-vaborbactam was as tolerable as comparators.

## 1. Introduction

Prompt and appropriate administration of antibiotics is key to treating infectious disease [1,2]. Carbapenems own remarkable anti-bacterial activity and remain first-line antibiotics in the treatment of patients with severe infections. However, the increasing incidence of carbapenem-resistance among Gram-negative bacteria (GNB), especially carbapenem-resistant Enterobacteriaceae (CRE) has limited the usefulness of carbapenems [3,4,5,6,7,8,9]. Hence, new effective antibiotics are urgently needed. Recently, several novel β-lactam/β-lactamase inhibitors, including ceftazidime-avibactam, ceftolozane-tazobactam, and meropenem-vaborbactam, have been developed to combat these multidrug-resistant organisms (MDRO) and have provided promising therapeutic options against Enterobacteriaceae-producing extended spectrum beta lactamases (ESBL), AmpC, and *Klebsiella pneumoniae* carbapenemase (KPC) [10,11,12,13,14,15]. Meropenem-vaborbactam is the first carbapenem-β-lactamase inhibitor combination to be approved in the USA for treating complicated urinary tract infections (cUTI), including acute pyelonephritis (APN). This novel antibiotic is a fixed-dose combination of meropenem—a carbapenem and vaborbactam—which is a new novel cyclic boronic acid-based β-lactamase inhibitor that can enhance the activity of meropenem [11,12]. Meropenem can inhibit the cell wall synthesis of bacteria by binding to penicillin-binding protein, and it alone has broad-spectrum activity, even for many antibiotic-resistant GNB. Despite vaborbactam alone having no anti-bacterial activity, it is a potent inhibitor of Ambler class A (KPC, CTX-M, SHV, and TEM) carbapenemases and class C (MIR and P99) β-lactamases [11,12]. In vitro, meropenem-vaborbactam combination has shown potent activity against mangy MDRO, including KPC-producing CRE [11,12,14,16]. However, clinical studies investigating the clinical efficacy of meropenem-vaborbactam in the treatment of infectious disease have been limited. To better understand the usefulness of meropenem-vaborbactam, this study reports the integrated analysis of two phase III studies [17,18] of meropenem-vaborbactam in the treatment of acute bacterial infections.

## 2. Methods

### 2.1. The Characteristics of Study

The Targeting Antibiotic Non-Susceptible Gram-Negative Organisms (TANGO) program comprised two phase III randomized, multicenter, multinational studies: TANGO I (NCT02166476) and TANGO II (NCT02168946) [17,18]. TANGO I included adult patients with cUTI or APN and compared the clinical efficacy and tolerability of meropenem-vaborbactam (2 g/2 g for 3-h infusion) or piperacillin-tazobactam (4 g/0.5 g for 30-min infusion) every 8 h for 10 days of total treatment (intravenous ± oral) [17]. TANGO II included adult patients with confirmed/suspect CRE infections (bacteremia, hospital-acquired/ventilator-associated pneumonia (HAP/VAP), complicated intra-abdominal infection (cIAI), and cUTI/APN) and compared the effect and safety of meropenem-vaborbactam (2 g/2 g for 3 h every 8 h for 7–14 days) and best-available therapy (mono or combination therapy with polymyxins, carbapenems, aminoglycosides, tigecycline, or ceftazidime-avibactam alone) [18].

### 2.2. Analysis Population and Outcome Measurement

The modified intent-to-treat (MITT) population was the population to assess adverse events and comprised all patients who received one or more doses of the study drug. The microbiologic MITT (mMITT) population included all patients in the MITT population who had a baseline-qualifying bacterial pathogen. Efficacy endpoints included the proportion of patients with clinical cure at end of treatment (EOT) and test of cure (TOC) (7 ± 2 days after EOT), and the proportion of patients with microbiologic eradication at EOT and TOC. Clinical cure was defined as the complete resolution of signs/symptoms of the index infection and microbiologic eradication was defined as the microbiologic eradication or presumed eradication (clinical cure in the absence of a sample for repeat culture). Overall success was defined as the composite outcome of clinical cure and microbiologic eradication.

### 2.3. Statistical Analysis

Categorical variables were reported as frequency counts with percentages. In addition, the differences of baseline characteristics between the meropenem-vaborbactam and control groups were evaluated using Pearson’s chi-squared test for categorical variables. Treatment effects, including clinical cure rate, microbiological eradication rate, and the risk of adverse events were calculated as a risk ratio (RR) with 95% CI for dichotomous data using a random-effects model.

## 3. Results

### 3.1. The Clinical Manifestations of Patients

Overall, a total of 421 patients (meropenem-vaborbactam group: 224 and comparator group: 197) were utilized in this analysis. Their mean age was 55.2 years and 35.6% (*n* = 15) of patients were ≥ 65 years. The percentage of patients who were female was 63.7% (*n* = 268) and 91.9% (*n* = 387) of patients were white. One hundred and thirty-seven patients (32.5%) had systemic inflammation response syndrome and 250 patients (59.4%) had a Charlson Comorbidity Index score ≥ 3. The most common type of infection was cUTI/APN (*n* = 378, 89.8%), followed by bacteremia (*n* = 22, 5.2%), HAP/VAP (*n* = 5, 1.2%), and cIAI (*n* = 4, 1.0%). *Escherichia coli* was the most common pathogen (*n* = 246, 58.4%), followed by *Klebsiella pneumoniae* (*n* = 99, 23.5%), *Proteus mirabilis* (*n* = 20, 4.8%), *Enterobacter cloacae* spp. (*n* = 18, 4.3%), and *Serratia marcescens* (*n* = 2, 0.5%). There was no significant difference in terms of the demographic and baseline characteristics between meropenem-vaborbactam and the control group (Table 1).

### 3.2. Clinical and Microbiological Responses

**Figure 1:**
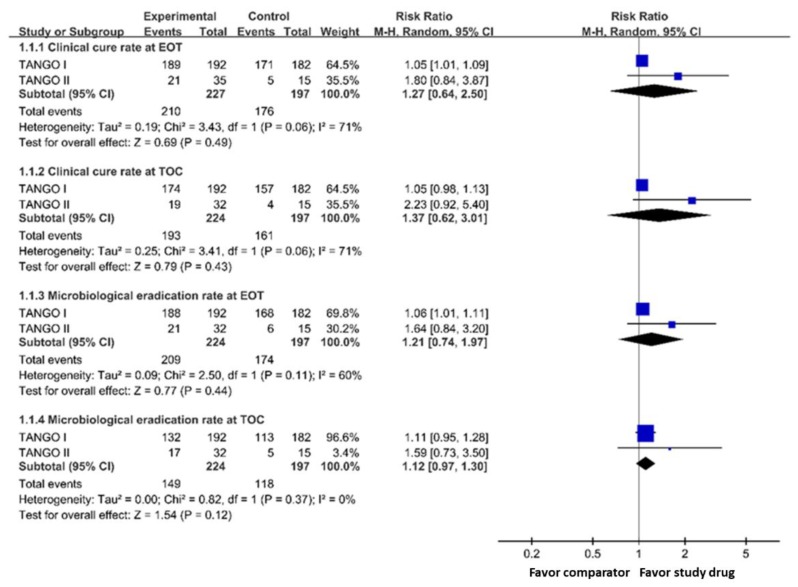
Clinical cure rate and microbiological eradication rate of meropenem-vaborbactam and comparator in microbiological modified intention-to-treat population analysis. Legend: EOT, end of treatment; TOC, test of cure.

The clinical cure rates at EOT and TOC among the meropenem-vaborbactam group were non-inferior to those of the control group (at EOT, 92.5% versus 89.3%, RR 1.27, 95% CI 0.64–2.50; at TOC, 86.2% versus 81.7%, RR 1.37, 95% CI 0.62–3.01) in the mMITT populations (Figure 1). In addition, meropenem-vaborbactam was non-inferior to comparators for microbiological eradication at EOT and TOC (at EOT, 93.3% versus 88.3%, RR 1.21, 95% CI 0.74–1.97; at TOC, 66.5% versus 59.9%, RR 1.12, 95% CI 0.97–1.30) (Figure 1). In the subgroup of patients with cUTI/APN, meropenem-vaborbactam had a similar overall success rate to the control group at EOT (RR 1.05, 95% CI 1.01–1.09) and at TOC (RR 1.05, 95% CI 0.93–1.19).

### 3.3. Safety

**Figure 2:**
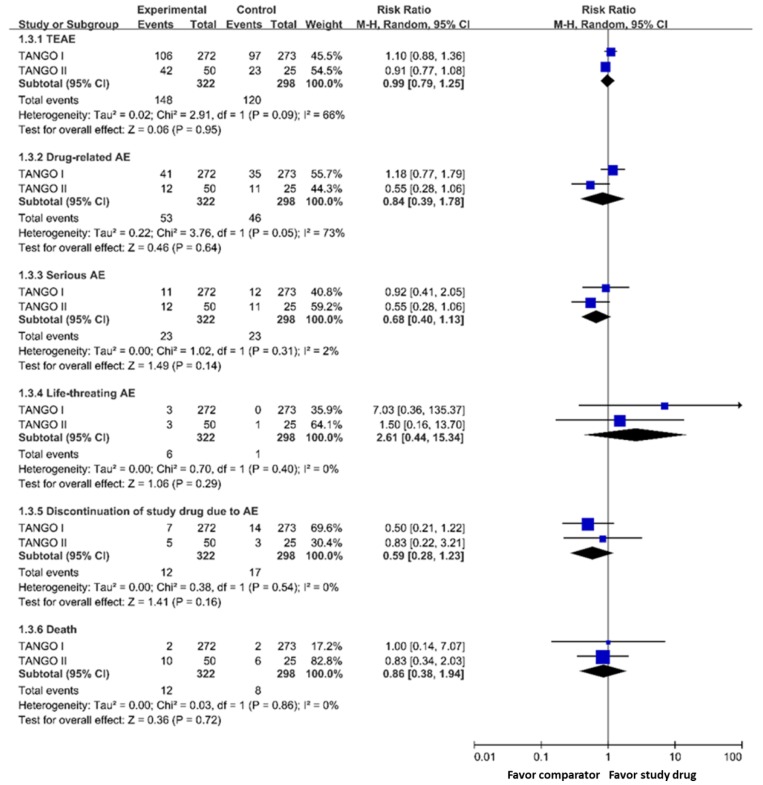
Risk of adverse events of meropenem-vaborbactam and comparators in modified intention-to-treat population analysis. Legend: TEAE, treatment-emergent adverse event; AE, adverse event.

Meropenem-vaborbactam had a similar risk of (i) treatment-emergent adverse events (RR 0.99; 95% CI 0.79–1.25), (ii) events leading to discontinuation of the study drug (RR 0.59; 95% CI 0.28–1.23), (iii) any serious adverse events (RR 0.68; 95% CI 0.40–1.13), (iv) life-threatening adverse events (RR 2.61, 95% CI 0.44–15.34), (v) drug-related adverse events (RR 0.84, 95% CI 0.39–1.78) when compared with the control group (Figure 2). In addition, meropenem-vaborbactam was associated with a similar risk of death when compared with the control group (RR 0.86; 95% CI 0.38–1.94) (Figure 2).

## 4. Discussion

In this integrated analysis of TANGO I and TANGO II, meropenem-vaborbactam was noninferior to comparators in the treatment of acute bacterial infections. The results of clinical efficacy were consistent between EOT and TOC assessment in the overall mMITT population. In addition, the meropenem-vaborbactam demonstrated a similar rate of microbiological eradication at both EOT and TOC to comparators in the integrated analysis. In the subgroup analysis of patients with cUTI/APN, the overall success rate of meropenem-vaborbactam was noninferior to comparators. This is consistent with other type infections which were only enrolled in the TANGO II trial [18]. Among patients with HAP/VAP or bacteremia, the 28-day all-cause mortality rate was lower in the meropenem-vaborbactam group than the comparator group (22.2% (4/18) versus 44.4% (4/9), difference, −22.2%; 95% CI −59.9 to 15.5%; *p* = 0.25)). Among the four patients with cIAI, the clinical cure rate at TOC was 100% (2/2) in the meropenem-vaborbactam group and 0% (0/2) in the comparator group. Overall, the integrated analysis of almost 421 patients demonstrated that meropenem-vaborbactam is as effective as comparators in the treatment of acute bacterial infection, particularly cUTI/APN.

In the safety analysis of the MITT population, we found that meropenem-vaborbactam had a similar risk of treatment-emergent adverse events, events leading to discontinuation of the study drug, any serious adverse events, life-threatening adverse events, and drug-related adverse events (RR 0.84, 95% CI 0.39–1.78) in comparison with the control group. The results were consistent with the risk of death analysis in that meropenem-vaborbactam was associated with a similar risk of death to comparators. Hence, this intergrade analysis demonstrated that meropenem-vaborbactam was safe and well-tolerated, with safety results similar to comparators.

This integrated analysis has two major limitations. Firstly, only a small sample was included in this analysis. Secondly, the number of CRE was limited. In the TANGO I trial [17], only three of the 57 *K. pneumoniae* isolates were resistant to meropenem, but none of the 239 *E. coli* isolates were resistant to meropenem. In the TANGO II trial, 47 isolates were found to be CRE. We therefore cannot evaluate the usefulness of meropenem-vaborbactam in the treatment of CRE infections. Third, this integrated analysis lacks the detailed characterization of pathogenic bacteria, such as types, subtypes, susceptibility to antibiotics, secretion of enzymes, and antibiotic-resistance mechanisms. Further large-scale and more detailed study is warranted to clarify our findings.

In conclusion, in this work meropenem-vaborbactam was found to be noninferior to comparators for clinical cure and microbiological eradication in the treatment of acute bacterial infection, particularly cUTI/APN. In addition, meropenem-vaborbactam was as tolerable as comparators and could be applied as another therapeutic option for acute bacterial infection.

## Figures and Tables

**Table 1:** Demographic characteristics for patients in the Targeting Antibiotic Non-Susceptible Gram-Negative Organisms (TANGO) I and TANGO II studies in the microbiologic modified intent-to-treat (mMITT) population.

Characteristics	Number of Patients (%)		*p* Value
	Meropenem-Vaborbactam (*n* = 224)	Comparator (*n* = 197)	
Age ≥ 65 years	70 (31.3)	80 (40.6)	0.059
Female	143 (63.8)	125 (63.5)	0.970
White	206 (92.0)	181 (91.9)	0.887
Creatinine clearance ≤ 50 mL/min/1.73 m^2^	103 (46.0)	93 (47.2)	0.882
Diabetes mellitus	44 (19.6)	41 (20.8)	0.854
Systemic inflammatory response syndrome	70 (31.3)	67 (34.0)	0.627
Charlson Comorbidity Index score ≥ 3	131 (58.5)	119 (60.4)	0.767
Type of infection			
cUTI/APN	194 (86.6)	184 (93.4)	0.077
Bacteremia	14 (6.3)	8 (4.1)	0.430
HAP/VAP	4 (1.8)	1 (0.5)	0.438
cIAI	2 (0.9)	2 (1.0)	0.690
Baseline pathogens			
*Escherichia coli*	128 (57.1)	118 (59.9)	0.629
*Klebsiella pneumoniae*	59 (26.3)	40 (20.3)	0.182
*Proteus mirabilis*	6 (2.7)	14 (7.1)	0.059
*Enterobacter cloacae* spp.	11 (4.9)	7 (3.6)	0.678

Legend: cUTI/APN, complicated urinary tract infection/acute pyelonephritis; HAP/VAP, hospital-acquired pneumonia/ventilator-associated pneumonia; cIAI, complicated intra-abdominal infection.
